# Low‐cost flexible thin‐film detector for medical dosimetry applications

**DOI:** 10.1120/jacmp.v15i2.4454

**Published:** 2014-03-06

**Authors:** P. Zygmanski, C. Abkai, Z. Han, Y. Shulevich, D. Menichelli, J. Hesser

**Affiliations:** ^1^ Department of Radiation Oncology Brigham and Women's Hospital, Harvard Medical School Boston MA USA; ^2^ Department of Radiation Oncology University Medical Center Mannheim, University of Heidelberg Germany; ^3^ IBA Dosimetry Bahnhofstrasse 5 90592 Schwarzenbruck Germany

**Keywords:** dosimetry, radiation detection, mechanical flexible and bendable detector, thin‐film photo diode, photovoltaic effect, direct X‐ray conversion

## Abstract

The purpose of this study is to characterize dosimetric properties of thin film photovoltaic sensors as a platform for development of prototype dose verification equipment in radiotherapy. Towards this goal, flexible thin‐film sensors of dose with embedded data acquisition electronics and wireless data transmission are prototyped and tested in kV and MV photon beams. Fundamental dosimetric properties are determined in view of a specific application to dose verification in multiple planes or curved surfaces inside a phantom. Uniqueness of the new thin‐film sensors consists in their mechanical properties, low‐power operation, and low‐cost. They are thinner and more flexible than dosimetric films. In principle, each thin‐film sensor can be fabricated in any size (mm^2^ – cm^2^ areas) and shape. Individual sensors can be put together in an array of sensors spreading over large areas and yet being light. Photovoltaic mode of charge collection (of electrons and holes) does not require external electric field applied to the sensor, and this implies simplicity of data acquisition electronics and low power operation. The prototype device use for testing consists of several thin film dose sensors, each of about 1.5 cm×5 cm area, connected to simple readout electronics. Sensitivity of the sensors is determined per unit area and compared to EPID sensitivity, as well as other standard photodiodes. Each sensor independently measures dose and is based on commercially available flexible thin‐film aSi photodiodes. Readout electronics consists of an ultra low‐power microcontroller, radio frequency transmitter, and a low‐noise amplification circuit implemented on a flexible printed circuit board. Detector output is digitized and transmitted wirelessly to an external host computer where it is integrated and processed. A megavoltage medical linear accelerator (Varian Tx) equipped with kilovoltage online imaging system and a Cobalt source are use to irradiate different thin‐film detector sensors in a Solid Water phantom under various irradiation conditions. Different factors are considered in characterization of the device attributes: energies (80 kVp, 130 kVp, 6 MV, 15 MV), dose rates (different ms × mA, 100–600 MU/min), total doses (0.1 cGy‐500 cGy), depths (0.5 cm–20 cm), irradiation angles with respect to the detector surface (0°‐180°), and IMRT tests (closed MLC, sweeping gap). The detector response to MV radiation is both linear with total dose (~1‐400 cGy) and independent of dose rate (100‐600 Mu/min). The sensitivity per unit area of thin‐film sensors is lower than for aSi flat‐panel detectors, but sufficient to acquire stable and accurate signals during irradiations. The proposed thin‐film photodiode system has properties which make it promising for clinical dosimetry. Due to the mechanical flexibility of each sensor and readout electronics, low‐cost, and wireless data acquisition, it could be considered for quality assurance (e.g., IMRT, mechanical linac QA), as well as real‐time dose monitoring in challenging setup configurations, including large area and 3D detection (multiple planes or curved surfaces).

PACS number: 87.56.Fc

## I. INTRODUCTION

Detection of ionizing radiation is essential in radiation protection, quality assurance (QA) and in medical applications involving imaging of anatomy and determination of dose distribution. In the latter cases, detection of ionizing radiation is typically accomplished with high‐resolution flat‐panel arrays,^1^, ^2^, ^3^, ^4^ low‐resolution ionization chamber/diode arrays,^5^, ^6^, ^7^ and intensifying screen/film systems.^8^, ^9^ Radiographic films or self‐developing films, such as GAFCHROMIC films, are flexible and can be cut to fit various geometries and are relatively inexpensive. However, they require postirradiation scanning and repeated calibrations and, in some cases, chemical processing, which significantly increases the overall cost of film dosimetry. Digital imagers or detector arrays, on the other hand, are mechanically rigid, heavy, and bulky. Modification of their architecture typically implies high development costs. Development of inexpensive and low‐tech digital devices, mechanically similar to films, for radiotherapy quality assurance (QA) is an attractive idea. In particular, the primary motivation of this study is the need for flexible arrays of thin‐film sensors as applied to radiotherapy dose verification in phantoms in multiple planes or curved surfaces.

In the last two decades, large area solar cells have become attractive as low‐cost alternative types for conversion of solar energy to electric power. Recent developments in thin‐film solar cells are becoming interesting since they offer both high flexibility (rolling radii of below 2 cm)^(10)^ and sensitivity to both optical and high energy photons.^(11)^ Besides their low cost and high mechanical flexibility, there are a wide range of production options available, such as the possibility to produce large areas (above 1 m^2^), as well as nanostructured electrode and active material layers (total thickness of about 0.5–100 μm), that may drive the process of integrating these systems into existing or new medical radiation devices.

Solar cells^1^, ^10^ are essentially photodiodes used for conversion of optical photons to photo‐current during solar illumination. A photocell can be use in photovoltaic or photoconductive modes. In an attempt to minimize the use of expensive semiconductor material in these devices, and thus reduce costs, thin‐film structures have been developed and are available as commercial products from several companies such as PowerFilmSolar (Ames, IA) and Nanosolar (San Jose, CA). There are several approaches to photovoltaic technology:^10^, ^11^, ^12^, ^13^, ^14^, ^15^, ^16^ thin‐film amorphous silicon (a‐Si) cells, organic photovoltaics (OPV), cadmium telluride cells (CdTe), or copper indium gallium selenide cells (CIGS). Compared to the conventional solid state detectors, thin‐film photovoltaics are thin, flexible, and adjustable to any shape or size and, in this respect, are similar to radiographic films. This suggests that thin‐film detector arrays based on thin‐film photovoltaics may be useful, where other detector types are not practical (e.g., due to their mechanical rigidity, design inflexibility, weight or size).

To the best our knowledge, the potential of flexible thin‐film photovoltaics with zero external bias (photovoltainc mode) has not been fully recognized and their properties not thoroughly investigated from the clinical application perspective. For instance, sensitivity of organic photovoltaic coupled with flexible phosphor layer to MV X‐rays has been only partially characterized.^(17)^ Thick (5 μm) film photovoltaics have been explored in combination with thin‐film transistor (TFT) backbone forming flexible keV xX‐ray imager^(15)^ operating under moderate external voltage (5‐20 V). Similarly, response of MV X‐ray detector utilizing polycrystalacm20311line CdTe photovoltaic thicker films (10‐1000 μm) have been simulated in combination with various metal plates to enhance the detector signal.^(18)^ However, until now, low‐cost flexible thin‐film photovoltaic sensors, potentially scalable to large‐area array of sensors, have not been experimentally tested in clinical radiotherapy beams.

In order to be able to assess the potential of thin‐film photovoltaic cells in the area of radiotherapy dose verification, we characterized the response of solar cells to radiation under different clinically relevant conditions. While this study focuses mostly on response of individual sensors to linac generated MV X‐rays, complementary data are presented for kVp X‐rays, as well.

## II. MATERIALS AND METHODS

### A. Detector

**Figure 1:**
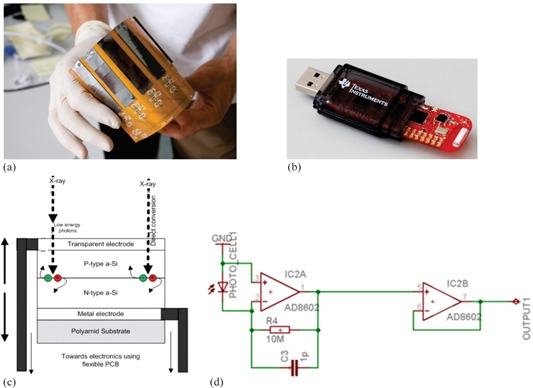
An array of eight sensors (a) mounted on flexible PCB; MSP430 USB‐receiver (b) for wireless data transmission; schematic diagrams of the principle of operation of thin‐film photocell (c); and the analog amplification circuit (d). X‐rays incident on thin‐film photocell can directly produce electron‐hole pairs, which are separated in the semiconductor junction and processed by additional amplification electronics. Transimpedance amplification and buffer circuit before digitalization of the separated charge of the photocell leads to a current in photovoltaic mode, which is amplified by a transimpedance amplifier.

The prototype detection system is composed of several flexible sensors with flexible readout electronics and a small data transmission module. Each sensor is a photoactive thin‐film a‐Si photocell (SP3‐13; PowerFilm Inc., Ames, IA) solar cell of about 1.5 cm×6.4 cm total area, 1.5 cm×5.0 cm active area, 200 μm total thickness, 3.0‐3.6 V operating voltage) (Fig. 1(a)). Smaller area photocells (e.g. 3 mm×3 mm) are possible to fabricate and use but, at the time of the study, only larger commercial cells were readily available. The specific photocells used in this study are based on a flexible thin‐film polyamide substrate. The a‐Si photo‐active material is deposited on the substrate in a roll‐to‐roll process by a vacuum vaporizer.^(16)^

The schematic of a photocell used in our readout system is depicted in Fig. 1(d). Raw signal is produced by direct electron‐hole pair generation from incident high‐energy photons.^(1)^ The photocells are use as direct X‐ray converters in the photovoltaic mode. Photovoltaic mode does not require external electric field and uses the inherent electric field resulting from different work functions of the electrodes. Employing photovoltaic mode simplifies the data acquisition circuit. This choice also minimizes the inner resistance of the system and leads to lower interference to electromagnetic noise generated by the linac. In general, small external electric field could be applied to the photocells to improve their sensitivity, but this was not pursued in our study. Because the photocell is very thin, conversion of X‐ray energy to an electrical signal (Fig. 1(c)) occurs without disturbing the dose distribution in the vicinity of the photocells. Each prototype detector is an array of dose sensors (of up to eight photocells), which can be independently read by a digital processing unit. In our study, multiple sensors are use to study the cell‐to‐cell variability of the dosimetric properties. Studying spatial distribution of dose with our prototype detection system and spatial resolution of 2D dosed distribution are beyond the scope of the paper. The photocells rest on a flexible printed circuit board (PCB); thus, the whole unit is flexible and we will refer to it as flexible thin‐film photovoltaic (FTF‐PV) detector, not to be confused with thin‐film transistors (TFT) used in readout electronics of the flat‐panel arrays.

Data acquisition system (Fig. 1(b)) uses USB microcontroller MSP430 (Texas Instruments, Dallas, TX) to digitize time‐dependent signal of each cell channel using a 10‐bit analog to digital converter (ADC). The digitized signal is sampled using different sampling frequencies and transmitted to a recording computer remotely using wireless radio‐frequency (RF) transmitter and USB receiver. The selection of MSP430 was motivated by availability, simplicity, and low‐cost of the microcontroller. The total cost for the prototype detector is about $120 USD, including photocells, analog processing electronics, digital ultra‐low power microcontroller, and radio‐frequency transmitter and flexible PCB on the foil.

### B. Data acquisition

The time‐dependent signal of each sensor channel $S_{i}(t)(i=1.8)$ is digitized by a 10‐bit analog to digital converter (ADC). The time‐dependent signal itself corresponds to the output voltage of the transimpedance amplification (Fig. 1(d)). The time‐dependent response of each detector cell is given by S(t), and when integrated over the whole irradiation time, it is denoted by

(1) $$S_{tot}=\sum_{n=1}^{N}S(t_{n})$$

where $t_{n}$ is the time of nth sample (n=1−N). The digitized signal $S_{i}(t_{n})$ is sampled using different sampling frequencies *f* The total response of each detector cell per irradiation is calculated in postacquisition filtering and integration of the raw signal. The dark currents are subtracted from the raw signal before integration. Photocells without light‐tight covering, had to be performed with the treatment room lights dimmed to minimize light incident on transparent electrode. Under these conditions, dark current was determined to be nearly zero and within one least significant bit (LSB) of the typical ADC noise. In the present experiment, treatment room lights were dimmed to decrease the impact of ambient light on the signal; however, this is not an intrinsic problem since the cells can be covered with nontransparent layer in future applications.

In most of the measurements described below, we measured instantaneous signal without hardware‐based integration with sampling rates of about 44 ms, 16 ms, and 3.7 ms. In the measurement of IMRT fields with low instantaneous dose rate we use a modified electric circuit, whose effective sampling rate was 1 Hz (integrating the signal for 985 ms and discharging for 15 ms). This modified system allowed us to measure very low dose rates (e.g., closed MLC) with greater precision and larger sampling time of 1000 ms.

In an attempt to characterize the intrinsic sensitivity and stability of thin‐film cells, we also performed measurements with Keithley Electrometer 642 (Keithley Instruments Inc., Solon, OH) directly connected to thin‐film photocell via TRIAX BNC cable, and measured both the integral charge during the whole irradiation and current as a function of irradiation time with a Cobalt source. Cobalt source provided a stable X‐ray source output.

Finally, to characterize energy response of sensors in the low‐energy beams in addition to the linac, we also used the Cobalt source and kVp X‐ray tube. We normalized detector response as a function of energy to the reference response of the Cobalt source (1.25 MeV). We compared sensor energy response to the response of the ionization chamber, as well as to newer and older type standard dosimetry diodes^(19)^ (stereotactic field detector (SFD) and Rikner diodes).

### C. Experimental setup

The experimental setup was comprised of the thin‐film photocells sandwiched between slabs of Solid Water and irradiated using 6 and 15 MV or 80 and 130 kVp kV beams of Varian TX. Similar irradiations were performed using A12 Exradin (PTW, Freiburg, Germany) ionization chamber. For determination of angular dependence, the gantry was rotated around the phantom. For most measurements, the FTF‐PV sensors and A12 ion chamber were placed at the midplane of the Solid Water phantom of 10 cm total thickness; the field size was c=30 by 30 cm^2^ and the detector was at SDD=100 cm. Measurement of depth profiles was performed with variable buildup.

Angular dependence and IMRT response were measured in 17 cm thick phantom with $6.5\times 6.5\;{\text{cm}}^{2}$ fields. Since the angular dependence measurements were measured in a rectangular phantom, the depth dependence for a particular gantry angle was removed by dividing the sensor signal by the ionization chamber signal.

Unfortunately, for the present prototype system the readout circuit and MSP430 transmitter were relatively close to the photocells and placing the whole device at the center of a large field to avoid the edge effects was not possible. In principle, this can be avoided by increasing the distances and making the readout electronics flatter. Instead, by selecting a smaller field size, the active area of the photocells was fully covered and the nearby electronics was located just outside of the field edge, which was the necessary compromise in the present experimental setup.

### D. Total and relative signal

The electronic readout circuit digitizes the output voltage of the transimpedance amplification, and the raw data correspond to sampled voltage information. This signal is not integrated in the hardware, except for IMRT measurements for which the modified system with an integration capacitor is used. Even in case with hardware‐based integration, the sampling time is only 1 s and requires postacquisition summation of the temporal signal. We achieved this by applying postacquisition filtering to determine the onset and end of radiation, and subsequent summing over the data points.

The units of S(t) and $S_{\text{tot}}$ are (ADC) and (ADC × number) of sampling points correspondingly. In addition, relative detector response for a particular cell is being used to characterize detector properties with respect to the reference conditions,

(2) $$R=S_{\text{tot}}/{\left(S_{\text{tot}}\right)}_{\text{ref}}$$

where $({S_{\text{tot}})}_{\text{ref}}$ is the total reference signal measured under specific reference conditions described in the text below. The relative detector cell response *R* depends on several varying parameters (m, r, c, d, e, g, ϕ, f, where m = monitor units or time, r = dose rate, c = rectangular field size, d = depth in Solid Water phantom, e = nominal energy of the source, g = dynamic MLC sweeping gap size, φ = gantry angle, f = sampling frequency). While a given parameter of interest is being changed, others are kept constant under certain reference conditions. Each of the parameters represents a factor important in radiotherapy. An overview of the parameters, their relevance, and their ranges are given in Table 1.

**Table 1:** Specification of variables, their relevance for radiotherapy and their values

*Variable*	*Description (Relevance)*	*(Values) Dimension*
m	monitor units or time (linearity of response)	(3.2−400) ms or (1, 2, 5, 10, 20, 50, 100, 200, 400) MU
r	dose rate (dynamic, time related effects)	(10−160) mA or (100, 200, 300, 400, 600) MU/min
c	rectangular field size (homogeneity of the device's sensitivity)	$(30\times 30,\;12\times 12,\;6.5\times 6.5)\;{\text{cm}}^{2}$
d	depth in solid water phantom (attenuation and phantom scatter properties)	(0.5, 1.0, 1.5, 3, 5, 10, 15, 20) cm
e	nominal energy of the source (energy dependence, beam quality)	(50, 70, 100, 120, 140, 150, 200, 250, 280) kVp or (1.25) MeV or (6, 15) MV
g	dynamic MLC sweeping gap size (dependence on instantaneous dose rate and on beam hardening)	0 (closed MLC) or (1, 5, 10, 20) mm
ϕ	gantry angle with respect to the normal to the surface of the detector (relevant for oblique irradiations)	(0° (perpendicular), 20°, 45°, 80°, 100°, 130°, 150°, 180°)
f	sampling frequency used to digitalize and record the data f (time resolution of the system)	(1, 23, 62, 268) Hz

## III. RESULTS

Results below are given for individual sensors (photocells). The array of sensors is not used here to characterize spatial dose distribution, but rather to determine cell‐to‐cell variability for the same irradiation conditions. All the following results are for 6 MV linacs, unless otherwise stated.

### A. Linearity

**Figure 2:**
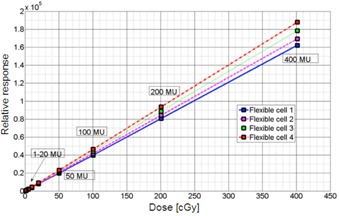
Total photocell response $S_{\text{tot}}(D)$ [ADC] vs. ionization chamber dose D [cGy]. The integrated ADC units scale linearly with applied dose, as measured by the reference ion chamber. As one can see. different detector cells have different linear response, which can be calibrated to rsult in dose (6 MV).

**Figure 3:**
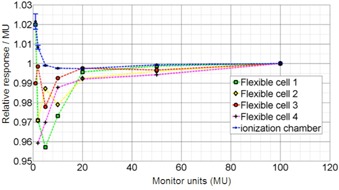
Normalized photocell and ion chamber response per monitor unit (R(m) / m) as a function of monitor unit $\left(m_{\text{ref}}=100,6\;\text{MV}\right)$.

The total signal of each sensor is linearly proportional to dose as seen in Fig. 2. The linear regression fit $\left(R^{2}=1-{\text{SS}}_{\text{resid}}/{\text{SS}}_{\text{total}}\right)$ for all cell is in the range of 0.9999‐1.000, where ${\text{SS}}_{\text{resid}}$ is the sum of the squared residuals from the regression, and ${\text{SS}}_{\text{total}}$ is the sum of the squared differences from the mean. The slope of $S_{\text{tot}}$ (D) for various cells differs only by a few percent, indicating that their sensitivities are comparable to each other. The ratio of R(m) and monitor unit m is represented in Fig. 3 to show in greater detail the responses for small number of monitor units (below 15 MU). All the data points are normalized to rsponse for $m_{\text{ref}}=100\;\text{MU}$. A small increase of R(m)/m for m =1,2 for the ion chamber may be attributed to higher linac output rather than ionization chamber response.

### B. Angular dependence

**Figure 4:**
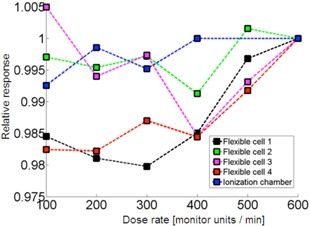
Normalized response $R_{\text{FTF}-\text{PV}}(\phi )/R_{\text{ion}}(\phi )$ as a function of the irradiation angle $\phi (\phi_{\text{ref}}=0^{\circ },6\;\text{MV})$.

The photocell response is normalized to the ion chamber response for $\phi_{\text{ref}}=0^{\circ }$ and depicted in Fig. 4. Both the photocell and ionization chamber have been sandwiched with the same Solid Water setup. However, one has to note that photocells are characterized by different geometrical shape, resulting in different active volume (very thin and large area). Furthermore, for ϕ 0° the beam penetrates the photocell first and then PCB, and vice versa for $\phi =180^{\circ }$. In essence, the design of the present photocell detector is not symmetric — with some differences between the front and back electrodes, as well as the back support. All these aspects could lead to significant differences in angular response especially for the posterior versus anterior and lateral beams, leading to more than 10% differences (e.g., for ϕ almost equal to 130°–180°), as well as to other uncertainties seen in Fig. 4.

Similar angular dependence observed for other type of detectors such as Matrixx (Iba Dosimetry America, Inc., Bartlett, TN) ion chamber array detector,^(5)^ which has a more complex internal structure. In case of ion chamber array, photon and electron transport through high‐Z materials and ion chambers differs for posterior versus anterior beams due to the forward and backscatter. Dose for the posterior beams is perturbed differently near their vicinity to impact Matrixx readings by about 10% compared to the anterior beams, which is very similar to the results we have observed for the FTF‐PV. Future FTF‐PV systems should, therefore, be realized on a thin plastic support layer to avoid the aforementioned AP‐PA asymmetry and integrated with the photocells directly on the PCB foil with minimal amount of high‐Z conductive paths or back‐structure.

### C. Changing dose rate

**Figure 5:**
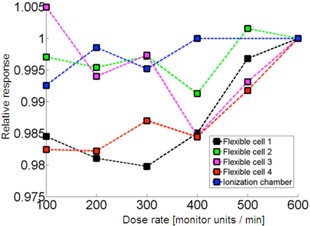
Dose rate dependence of photocells in comparison to ionization chamber reference measurements. The responses are normalized to dose rate $r_{\text{ref}}=600\;\text{MU}/\min$ (6 MV).

In Fig. 5, the dose rate dependence R(r) of the photocell detector is depicted for various cells for the same dose. All the data points were normalized to rsponse for $r_{\text{ref}}=600\;\text{MU}/\min$. Notably, some cells perform better than the others for the same irradiation conditions. Variation of signal in some cells is only within about ±1% and others reveal up to 2% drift.

Manufacturing reproducibility of photocells is not as good as standard diodes used in dosimetry. However, our results show that, with gain and dark current calibration, this variability can be corrected. The causes of differences between individual cells and between them and ionization chamber can be explained as follows.

To evaluate the accuracy of the proposed prototype system for high and low doses, detector response to monitor unit ratio (R(m)/m) is given, as well (Fig. 3). An optimal detecting system would have the same accuracy also for low monitor units. Discrepancy between the ionization chamber and the photocells is seen for m<10. However one has to consider that for m<5 accurate dosimetry starts to be difficult even when ionization chamber is used. The error due to accuracy of the linac output and ion chamber measurement is limited in low monitor unit region (linac output bias and fluctuations and increased ionization chamber leakage and noise). It needs to be observed that for low‐MU irradiations, the electrometer readout is low and is limited by about ±1 pC inherent uncertainty, which is represented by the error bars in Fig. 3. Thus the estimated error of the ionization chamber is within about ±0.5% just due to the noise inherent to electrometer readings. The dose measured by our prototype system has, however, an accuracy of about 1%‐3% for low (e.g., m ~ 1) monitor units, which is equal to about 0.01‐0.03 cGy, which is still a very small dose in the absolute sense in many measurements. The accuracy is worse for some cells and better for others, which might be due to the differences in their manufacturing. Another plausible reason of dose discrepancy could be the interplay between linac pulses and sampling rate.

### D. Depth dependence

**Figure 6:**
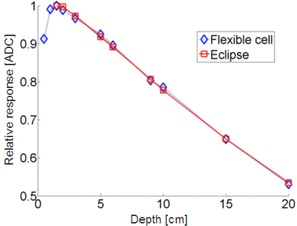
Attenuation profile R(d) with $d_{\text{ref}}=1.5\;\text{cm}\;(\text{SDD}=100\;\text{cm})$ for measured response vs. dose calculated by Eclipse TPS (6 MV).

The results are compared to doses integrated over the area of the photocell based on relative dose distribution from the planning system and are presented in Fig. 6. The deviations in dose depth dependence are up to 1%. In the prototype FTF‐PV, the photocells were located close to the microcontroller and, therefore, they could not be placed at the center of the Solid Water phantom, but rather at the phantom edges. Furthermore, because individual sensors used in the current study had rather large active area (1.5 cm×5.0 cm), in some cases, dose for each one of them was compared to average dose within the photocell area calculated by the treatment planning system. The relative dose distribution in the plane of the measurement determined from a clinically commissioned Eclipse (Varian Medical Systems, Palo Alto, CA) treatment planning system (TPS), using analytical anisotropic algorithm (AAA), algorithm was used to calculate an average dose to the detector cell area of the given cell only when point measurements with ionization chamber were insufficient to accurately determine the absolute dose to the total cell area. For instance, this was done when specific difficult irradiation conditions resulted in a dependence of area dose‐on‐dose gradient, such as during depth‐dependence measurements. Based on our commissioning, the uncertainty of dose calculation in TPS was about ±1%−1.5% within the field away from the field edge.

### E. Time dependence of the raw signal

**Figure 7:**
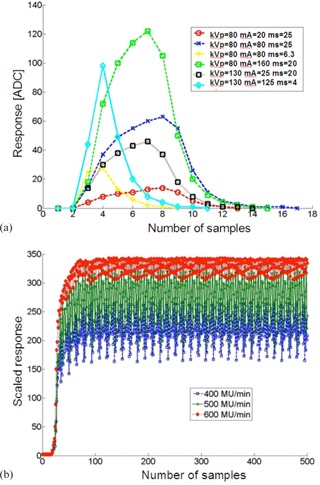
Raw data (a) for 80 kVp and 130 kVp and various values of mAs. The maximal sampling frequency in the current system is applied here, because the pulses are within 4‐25 milliseconds; time dependence of sensor signal (b) for 6 MV and m=50 and different dose rates. The raw data was rescaled to show differences in the Moiré‐aliasing patterns. Sampling rate was f=268Hz in all cases.

The raw detector response as a function of time S(t) is depicted in Fig. 7 for kV and MV energies, as well as short (fraction of a second) and long (one minute) irradiation times. It can be observed that for short duration of X‐ray kV output there is a characteristic increase and decrease of the output, but the signal is smooth (Fig. 7(a)), while for MV beam, the interference patterns (specifically Moiré‐aliasing) can be observed between the sampling rate of the photocell and the linac pulses (Fig. 7(b)).

This can be explained by noting that linac generates radiation in packets of pulses. Each packet of microsecond duration is separated by pauses of millisecond duration. Each microsecond packet contains many short picosecond pulses. Because the sampling rate of our prototype is limited by the wireless and ultralow power device specifications, the system has limited sampling rate of about f=268 Hz. With insufficient sampling rate, some of the packets (pulses) are missed.

**Figure 8:**
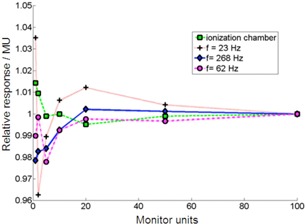
Normalized photocell and ion chamber response per monitor unit (R(m,f) / m) as a function of monitor unit $\left(m_{\text{ref}}=100,6\;\text{MV}\right)$. Different sampling frequencies affect the total sensibility of the detector cell. Dose rate was $r_{\text{ref}}=400\;\text{MU}/\min$.

In Fig. 8, dependence of response on monitor units for various sampling frequencies R(m,f) are seen, which highlight the relation of photocell response to sampling. For smaller irradiation times (smaller MU) and smaller frequency, more variations between the photocell signal and the ionization chamber signal could be observed, which can be explained by the under sampling (aliasing) problem, as shown in Fig. 7(b).

2, 7 show that in MV beams, a significant oscillation of the raw signal (without hardware integration) is observed. This effect is caused by the interplay between the high frequent linac pulses and photocell sampling (beats). This leads to the small, though noticeable, uncertainties such as those seen in 5, 7. Hardware integration with short integration period (e.g., ≤ 1 s) solves this specific problem.

### F. Energy dependency for kV and MV

The photocells have different energy response in MV range than in kV range. However, for a given kVp or MV beam, gain correction factor can be applied. Specifically, when the same dose is measured in reference conditions with the ionization chamber in 6 MV and 15 MV beams (D(6 MV) / D(15 MV) = 0.95) the photocell response ratio is $S_{\text{tot}}(6\;\text{MV})/S_{\text{tot}}(15\;\text{MV})=0.97$. This energy dependence in 6‐15 MV range of the raw integrated signal of FTF cell is acceptable, considering that calibration for each nominal linac energy is carried.

**Figure 9:**
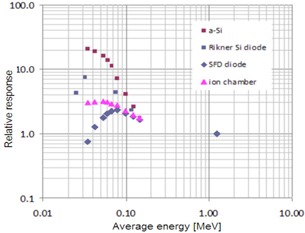
Energy dependence for various detectors including a‐Si FTF‐PV normalized to the Cobalt source energy 1.25 MeV Energies in keV range represent average energies of kVp spectra (integrated energy fluence divided bv integrated fluence) generated by an X‐ray tube.

Energy response for kV sources normalized to Cobalt energy is depicted in Fig. 9. In kV range the energy dependence of the photocells is significantly larger than in MV range, as expected based on photoelectric cross‐sectional dependence on energy. Response of photocell is greater than that of the ionization chamber. The response of the standard Rinker diode dosimeter reveals larger sensitivity to energy, as well. These results are also comparable to those reported in literature (e.g., for energy dependency in radiochromic films for MV and for kV / MV^20^, ^21^, ^22^).

### G. IMRT fields and low dose rate

**Figure 10:**
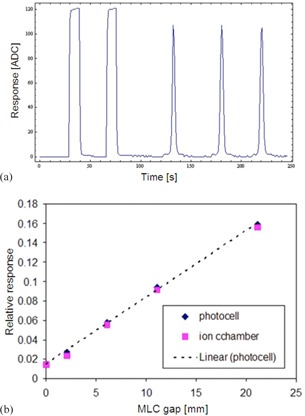
Raw signal of FTF‐PV (a) in hardware‐integration mode for two open beam and three 20 mm sweeping gap irradiations: $e_{\text{ref}}=6\;\text{MV},r_{\text{ref}}=400\;\text{MU}/\min,m_{\text{ref}}=100\;\text{MU}$ and 200 MU, 6 MV; response (b) of sweeping gap and closed MLC as a function of gap size. For closed MLC pattern, gap size was assumed to be zero.

In order to obtain more precise measurements in low‐dose conditions such as IMRT fields and closed MLC fields, photocells were use in hardware‐integration mode (charge accumulation) with 1 s sampling time. An example of photocell response to consecutive two irradiations of open beam and three sweeping gap patterns (with MLC gap g=20 mm) is shown in Fig. 10(a). The resulting relative responses to sweeping gap fields and closed MLC are shown in Fig. 10(b). In plotting data in Fig. 10, it was assumed that the closed MLC can be ascribed to zero MLC gap and that the signals are normalized to open beam signals (g→∞).

During IMRT delivery, both the dose rate and spectrum of the beam at any given point of measurement may change. Specifically, the beam is hardened (the low energy photons are attenuated) and the effective instantaneous dose rate is lowered (by up to about 60 times for closed MLC compared to the open beam value). Based on sweeping gap data such as seen in Fig. 10, we can conclude that the FTF‐PV detector is performing reasonably well under very low dose conditions.

### H. stability and sensitivity

Stability and sensitivity of photocells were determined with a photocell directly connected to an electrometer without prior amplification of the raw signal and irradiations with a stable output source (Cobalt source). The current drifts were observed during the initial irradiation of the photocell by about 2%; however, after reaching about 500 cGy of total dose (warm‐up dose), it stabilizes below the noise level. Long irradiations indicate 0.5% stability within 100 min. The initial warm‐up dose of about 500 cGy and stability of FTF are comparable to ionization chamber properties.

The current, measured with an electrometer for a given irradiated dose and area of the detector cell, allows determination of sensitivity per unit area and active thickness of the photocells. With the dose rate of 1 Gy/min, an active area of the detector cell about 645 mm^2^, and measured currents about 310 pA, the sensitivity per unit area is $s_{\text{area}}$ almost equal to 30 pC/Gy mm^2^. Assuming that the mean ionization energy of a‐Si is $E_{i}=4.4\;\text{eV}$ and its density is $\rho =2.3\;g/{\text{cm}}^{3}$, the expected sensitivity per unit volume is $s_{\text{volume}}$ almost equal to $q.\rho /E_{i}$ almost equal to 520 nC / Gy. mm^3^. From this, it follows that the active thickness of FTF‐PV is about $w=s_{\text{area}}/s_{\text{volume}}$ almost equal to 50 nm.

As a reference, the sensitivity of a‐Si photodiodes typically used in EPID X‐rays imagers (for instance, Perkin Elmer XRD1640 used in direct detection configuration) is measured as sA almost equal to 0.4 nC/(Gy·;mm^2^). This corresponds to an active thickness of a‐Si of about 800 nm. The difference in active thickness between the EPID and FTF‐PV is explained below.

The difference in active thickness between the EPID (800 nm effective thickness) and FTF‐PV (50 nm) cell can be explained by different physical thickness, different diffusion length of minority carriers, which is in turn related to material quality, or specific physical structure (single or multiple junction) and chemical composition (doping), which are optimal for conversion of visible light to solar energy and not necessarily for conversion of ionizing radiation to current. It is also possible that the off‐the‐shelf solar cell deposition process is not optimized to have a larger diffusion length of minority carrier because such solar cells are not meant for other than daylight solar power applications.

The present study was limited to testing of individual sensors (single pixels). Based on the current results, two‐dimensional flexible thin‐film arrays would be possible with pixel size of about 5 mm×5 mm (estimate based on our calculation of the sensitivity per unit area of $s_{\text{area}}$ almost equal to 30 pC/Gy. mm^2^). Thus the future developments in this area should address efficient FTF‐PV electronic design and dependence on pixel size of 2D flexible thin‐film (FTF‐PV) arrays.

To increase the external quantum efficiency of the FTF‐PV detection system, one can also combine the semiconductor layer with a scintillator, which converts the incident X‐ray to a low‐energy optical wave length, which is usually detected with higher internal quantum efficiency by the photo‐electric layer. To avoid high‐Z materials, various plastic scintillating materials are proposed in literature.^(23)^ This option is not considered in this paper in order to have a very thin and flexible system with linear response to radiation. However, in principle, depositing thin plastic scintillator on top of the transparent electrode of the thin film photovoltaic cell is feasible. Such a system might have all the desired properties and would be more sensitive to radiation than the present prototype. Our preliminary tests for thin‐film photodiode with 1 mm plastic scintillator showed an increase of signal by a factor of about 15 for 6 MV and 2 for kV beams.

## IV. DISCUSSION

### A. General

Dosimetric characteristics of the FTF‐PV cells are very promising, considering that the photocells were not optimized for X‐ray detection and that the readout electronics was very simple. Signal of each sensor is acquired as a function of time by an inexpensive USB microcontroller (MSP430) commercialized for wireless home applications. Dependence of the photocells on the major clinically relevant quantities is quite similar to that of the standard dosimeters (ionization chamber), even though the sensitivity per unit area of aSi thin film is smaller than the sensitivity of the standard diode detectors used in ionizing beams. This is mainly due to the nanometer thickness of FTF‐PV. With the improved electronic design, a larger amplification of the raw signal is expected. In addition, it is foreseeable that the quality of photocell fabrication can be improved by customizing its design for ionizing beams. Other photovoltaic materials can be considered for X‐ray detection — for instance, organic photovoltaics — which are under development and whose efficiency is increasing every year. The flexible thin‐film system described in this work is promising as a platform for prototyping quality assurance devices in radiotherapy. By providing distributed, low‐voltage power supply and wireless radiation sensing, the device can be easily integrated in clinical conditions.

### B. Potential applications

Due to the nanometer thickness of FTF‐PV cells and their flexibility, they can be use for in phantom dosimetry at multiple planes or curved surfaces. Dosimetry in water medium using FTF‐PV was not tested; however, assuming that the whole sensor array including the flexible PCB is covered with a thin layer (polyamid), application of FTF‐PV in water environment is foreseeable. Similarly, *in vivo* dosimetry was not tested, but the results here indicate that these sensors can be calibrated to measure the skin dose *in vivo.* A natural question in skin dose measurement would be sensitivity to electron contamination of the MV photon beams. If one wishes to keep the production cost of FTF‐PV arrays at low level (as noted herein), then this limits their spatial resolution to a few millimeters and thus precludes imaging of patient anatomy with millimeter or submillimeter spatial resolution. Thus, FTF‐PV as portrayed herein cannot compete with high‐spatial resolution (high‐tech and high‐cost) EPID detectors. On the other hand, commercially available ion chamber (Matrixx) or diode (MapCHECK, Delta^4^, OCTAVIUS) arrays used in IMRT QA have resolution ranging about 5‐7 mm. Therefore, because this level of spatial resolution is practically attainable, FTF‐PV arrays can be fabricated for IMRT QA. Finally, since the photocells are sensitive to MV as well as kV X‐rays and visible light, one might explore their usage for mechanical linac QA — for instance, in MV radiation vs. light vs. on‐board kV imaging isocenter. In the latter case, sub‐mm (about 200 μm) spatial resolution can be achieved by stacking FTF‐PV sensors together to form a linear array.

## V. CONCLUSIONS

Unique low‐cost, flexible, and wireless dose detector was demonstrated as a basis for further prototype work. To the best of our knowledge the device is the first mechanically flexible X‐ray digital detector. The device characteristics for measurement of ionizing radiation are very promising, considering extremely low cost and easiness of realization of the prototype device. Further developments should be carried in the area of optimization of thin‐film photocell structure and readout electronics.

## ACKNOWLEDGMENTS

The project was partially supported by JCRT grant. We want to express out gratefulness to Robert Cormack and Yulia Lyatskaya for providing helpful comments.
